# The Effects of Bariatric Procedures on Bowel Habit

**DOI:** 10.1007/s11695-016-2100-9

**Published:** 2016-02-19

**Authors:** Sorena Afshar, Seamus B. Kelly, Keith Seymour, Sean Woodcock, Anke-Dorothee Werner, John C. Mathers

**Affiliations:** 1 Northumbria Healthcare NHS Foundation Trust, North Shields, Tyne and Wear UK; 2 Newcastle University, Newcastle-upon-Tyne, Tyne and Wear UK

**Keywords:** Bariatric surgery, Bowel habit, Gut transit, Bristol stool form

## Abstract

**Background:**

Bariatric procedures are increasingly being used to combat the rising obesity epidemic. The aim of this study was to assess the effect of these interventions on bowel habit.

**Methods:**

We recruited obese adults listed for a bariatric procedure. Demographic data, medical history, medications and anthropometric measurements were recorded. Bowel habit was characterized using a 7-day Bristol Stool Form Scale (BSFS) diary. A validated food frequency questionnaire (FFQ) was used to assess diet.

**Results:**

Twenty-six patients were assessed pre-operatively and at a median of 6.4 months post-operatively. Nineteen had a Roux-en-Y gastric bypass (RYGB), five had a sleeve gastrectomy (SG) and two had an intra-gastric balloon (IGB) with median percentage excess weight loss (% EWL) of 67.9, 52.4 and 31.3 %, respectively. Dietary fibre intake decreased from 24.4 (±12.1) g/day pre-operatively to 17.5 (±7.3) g/day post-operatively (*P* = 0.008). Frequency of bowel motions decreased from 8.6 (±3.5) to 5.7 (±3.5) motions/week (*P* = 0.001). Mean usual BSFS score decreased (towards firmer stool) from 4.1 (±1.3) pre-operatively to 3.1 (±1.9) post-operatively (*P* = 0.016). Constipation increased from 8 to 27 %, but this did not reach statistical significance (*P* = 0.125).

**Conclusions:**

Constipation is a common problem after bariatric surgery. The decrease in bowel motion frequency and change towards firmer stools suggest prolonged intestinal transit time after bariatric procedures. Reduction in dietary fibre intake is likely to be a contributory factor.

## Introduction

Worldwide prevalence of obesity is increasing. Obesity is a major risk factor for common chronic diseases, including diabetes, cardiovascular diseases and cancer [1]. Whilst lifestyle-based interventions are the backbone of most public health strategies to prevent and treat obesity, bariatric procedures are increasingly being used to combat this rising epidemic. These interventions have significant benefits in terms of achieving sustained weight loss and improvement of obesity-related comorbidities [2]. However, disordered bowel habit appears to be an associated problem in at least a subset of patients [3], and this can have a significant negative impact on the patient’s quality of life [4, 5]. Obesity itself is associated with disordered bowel habit with most studies showing increased prevalence of diarrhoea, but not constipation in obese patients [3, 6]. This increased prevalence of diarrhoea is likely to be multifactorial and is likely to be related to diet. One hypothesis is that high intake of sugars and other products in excess of absorption capability leads to an osmotic diarrhoea [1].

The effects of specific bariatric procedures on bowel habit are not well defined. Some reports have suggested that the Roux-en-Y gastric bypass (RYGB) and biliopancreatic diversion (BPD) result in diarrhoea [7, 8]. In contrast, others have reported improvement in symptoms of loose stools after RYGB [9]. Most reports associate the adjustable gastric band (AGB) and sleeve gastrectomy (SG) with constipation [7, 8]. These inconsistencies may in part be due to the variety of validated and non-validated assessment tools used [3].

Bariatric procedures are likely to have procedure-specific effects on dietary intake as well as bowel habit. Therefore, to interpret the effects of such interventions on bowel habit, it is important to have longitudinal studies using validated assessment tools and robust information on dietary intake. There is a lack of such studies in the literature.

Therefore, as part of the observational study Biomarkers of Colorectal cancer After Bariatric Surgery (BOCABS study ISRCTN95459522), we assessed participants’ dietary intake and bowel habits before and after bariatric procedures.

## Methods

We recruited obese adults listed for a bariatric procedure at a single centre (North Tyneside General Hospital, North Shields, UK). Exclusion criteria were age <18 or >65 years, inflammatory bowel disease, previous weight loss surgery, previous colorectal resection, painful anorectal pathology (including anal fissure and symptomatic haemorrhoids) and pregnancy. Informed written consent was obtained from all individual participants included in the study.

Demographic data including age, sex, medical history and current medications were recorded for all participants. Anthropometric measurements including height, weight and waist and hip circumference were made using a standardised protocol by a single observer (SA). Percentage body fat was estimated using a bioimpedance device (Tanita TBF-300MA Body composition analyser). Data was collected pre-operatively and repeated at 6 months post-operatively. All participants underwent digital rectal and rigid sigmoidoscopic examination to 15 cm from anal verge at each time point. Significant macroscopic abnormalities including malignancy, polyps >1 cm and proctitis were planned for exclusion. A single random rectal biopsy, taken at each examination, was sent to a histopathologist for examination to exclude microscopic colitis.

### Bariatric Procedures

The RYGB involved laparoscopic formation of a 50-ml gastric pouch with a 100–150-cm alimentary limb and 60–75-cm biliopancreatic limb. Alimentary limb length was determined by the patients’ BMI and comorbidities (150 cm for BMI > 50 kg/m^2^ or in the presence of type 2 diabetes mellitus and 100 cm otherwise). Biliopancreatic limb length was chosen based on surgeon preference. The SG involved laparoscopic resection of the greater curvature of the stomach over a 34–36 F bougie. The intra-gastric balloon (IGB) was placed endoscopically (Allergen®) and filled with 500–700 saline mixed with 10 ml of methylene blue.

### Bowel Habit

Bowel habit was characterized using a 7-day Bristol Stool Form Scale (BSFS) diary. This tool has been validated and has been shown to correlate with whole gut transit time (WGTT) [10–12]. Usual BSFS score was defined as the most commonly reported score during the diary period (mode). We defined constipation as fewer than three bowel motions per week with lumpy or hard stools (BSFS 1 or 2) in at least 25 % of bowel motions recorded during the 7-day diary. Diarrhoea was defined as loose (mushy) or watery stools (BSFS 6 or 7) occurring in at least 75 % of stools. These definitions are in keeping with the Rome III criteria for diagnosis of functional gastrointestinal disorders [13]. Ambiguous diary entries were clarified by contacting the participants.

### Dietary Assessment

A validated food frequency questionnaire (FFQ) used in the European Prospective Investigation into Cancer and Nutrition (EPIC) study [14] was used to estimate the participant’s usual dietary intake. The FFQ was modified to assess intake over the previous 3 months and not the previous year as per original questionnaire, and tailored slightly to include foods eaten commonly in the north-east of England.

### Statistical Analysis

Data was collected in paper form and transferred to a study-specific database. Statistical analysis was carried out using SPSS software (Version 22.0 for Windows, SPSS, Chicago, USA). The Shapiro-Wilk test was used to test for normality of distribution of variables. Data is reported as mean ± standard deviations or median and interquartile range (IQR) as appropriate. Wilcoxon signed ranks, McNemar and paired sample *t* tests were used as appropriate. Cross-tabulation was carried out using exact tests (Monte-Carlo simulation). ANOVA or Kruskal-Wallis tests were used to compare the different procedure groups as appropriate. Spearman’s correlation coefficient was used to assess the strength of association between variables. Statistical significance was set at *P* < 0.05.

## Results

Thirty-eight candidates for a bariatric procedure were recruited; 32 (84.2 %) completed a BSFS diary at a median of 33 days pre-operatively (IQR 37.5).

Twenty-six out of the 32 (76.5 %) also completed a repeat diary at a median follow-up of 6.4 months (IQR 1.1) after the bariatric procedure. Therefore, 26 participants with paired data were suitable for inclusion. Participants had all successfully completed a 12-week weight management programme and achieved at least 5 % body weight loss prior to enrolment.

Table 1 shows the characteristics of the study participants. There was no statistically significant difference in age and sex distribution between the treatment groups (*P* = 0.809 and *P* = 0.260, respectively). RYGB patients achieved the most weight loss, followed by SG and IGB with percentage excess weight loss (% EWL—calculated based on ideal body weight of BMI 25 kg/m^2^) of 67.9, 52.4 and 31.3 %, respectively (*P* = 0.043). There were no cases of significant anorectal pathology or microscopic colitis.

**Table 1:** Participant characteristics

	Surgical procedure			
	RYGB *N* = 19	SG *N* = 5	IGB *N* = 2	*P* value
Age (years)–median (IQR)	49.0 (7.8)	44.6 (26.8)	49.1	0.809
Sex				
Female (%)	84.2	80	50	0.260
Male (%)	15.8	20	50	
Comorbidities *N* (%)				
Type II DM	5 (26)	1 (20)	1 (50)	0.717
Hypertension	4 (21)	1 (20)	2 (100)	0.053
Hyperlipidaemia	2 (11)	1 (20)	1 (50)	0.322
Previous cholecystectomy	3 (16)	1 (20)	0	0.799
Weight (kg)				
Pre-operative	114.8 (±17.6)	111.6 (±12.2)	131.9 (±11.9)	0.338
Post-operative	87.0 (±17.6)	86.3 (±12.3)	111.5 (±3.2)	0.148
BMI (kg/m^2^)				
Pre-operative	41.8 (±6.5)	40.5 (±1.9)	46.7 (±2.3)	0.453
Post-operative	31.7 (±5.7)	31.5 (±3.5)	40.1 (±0.6)	0.117
Percentage body fat				
Pre-operative	48.2 (±4.9)	50.5 (±0.8)	59.5 (±8.7)	*0.013
Post-operative	36.8 (±7.4)	39.1 (±5.2)	49.9 (±2.8)	0.052
% EWL–median (IQR)	67.9 (26.6)	52.4 (32.2)	31.3	*0.043

*RYGB* Roux-en-Y gastric bypass, *SG* sleeve gastrectomy, *IGB* intra-gastric balloon, *EWL* excess weight loss, *BMI* body mass index, *DM* diabetes mellitus

*Denotes statistically significant difference between the treatment groups (Kruskal Wallis test)

### Dietary Intake

Table 2 shows intake of relevant dietary factors pre- and post-operatively. As expected, daily energy intake decreased (*P* = 0.023). There is also a statistically significant difference in fibre intake, decreasing from 24.4 (±12.1) g/day pre-operatively to 17.5 (±7.3) g/day post-operatively (*P* = 0.008). Water, calcium and fat intake did not change significantly. Mean fibre intake was below the newly published Scientific Advisory Committee on Nutrition (SACN) recommended daily intake of 30 g/day at both the pre- and post-operative stage [15]. Only 38 % (10 out of 26) and 15 % (4 out of 26) of participants met this recommended intake level at the pre- and post-operative stage, respectively. This pattern was similar for all treatment groups.

**Table 2:** Dietary intake

	Pre-operative, *N* = 26	Post-operative, *N* = 26	*P* value
Energy (kcal/day)	1947 (±669)	1634 (±565)	*0.023
Fibre intake (g/day)	24.4 (±12.1)	17.5 (±7.3)	*0.008
Water intake (L/day)	2.5 (±1.2)	2.3 (±0.8)	0.220
Fat intake (g/day)	64.3 (±25.8)	59.2 (±22.3)	0.301
Calcium intake (mg/day)	757 (±261)	674 (±304)	0.232

*Denotes statistically significant difference between the pre- and post-operative groups

### Frequency of Bowel Motions

**Fig. 1:**
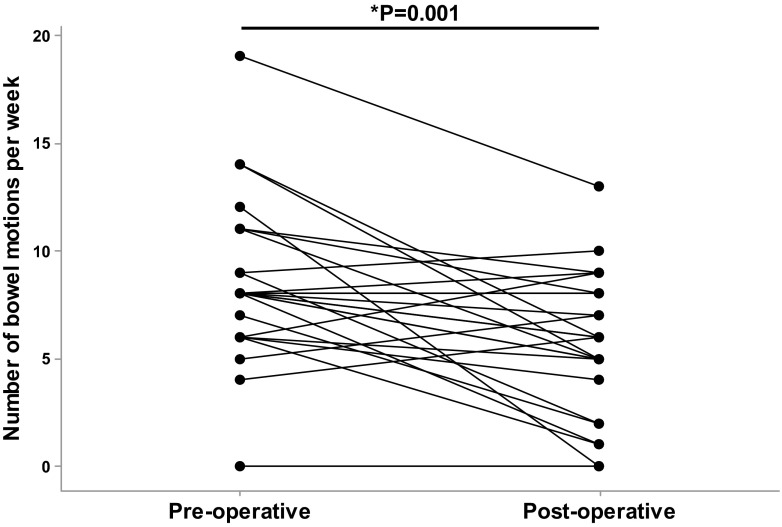
Frequency of bowel motions pre-operatively and at 6 months post-operative follow-up

Interindividual variation in bowel motion frequency was very large pre- and post-operatively (Fig. 1). At 6.4 months after surgery, mean frequency of recorded bowel motions decreased from 8.5 (±3.7) to 5.7 (±3.3) motions/week (*P* = 0.001). This change in frequency of bowel motions was not influenced by the bariatric procedure performed (*P* = 0.149). There was no significant correlation between any of the anthropometric measures or dietary factors and the change in frequency of bowel motions. There was no significant correlation between the change in frequency of bowel motions and alimentary or biliopancreatic limb length (Spearman correlation coefficients; *r* = 0.184, *P* = 0.480 and *r* = −0.168, *P* = 0.520, respectively. Data from 14 out of 19 RYGB cases; others missing data).

### Stool Form

**Fig. 2:**
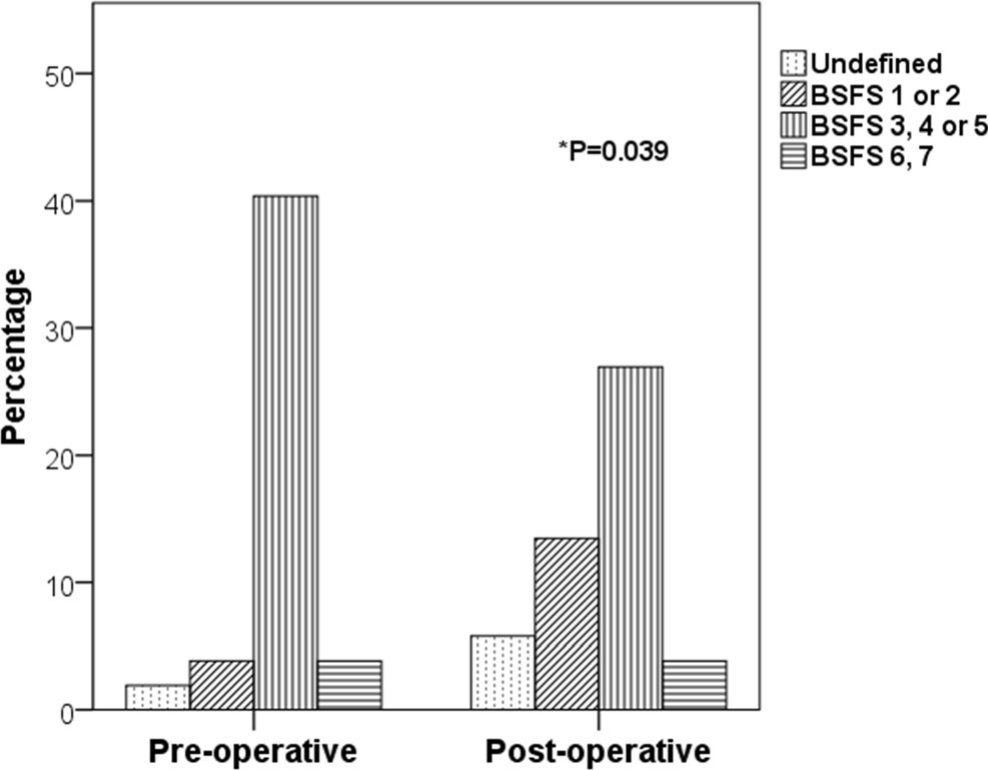
Bristol Stool Form Scale groups before and after bariatric procedures

Table 3 shows the distribution of stool forms estimated using the usual BSFS pre- and post-operatively. Data from a large prospective study of a general population sample is shown for comparison [12]. At 6 months follow-up, 15 out of 26 patients had a lower usual BSFS compared with that pre-operatively, four had a higher score and there was no change in the remainder (*P* = 0.028). Figure 2 shows the usual BSFS as analysed in four distinct groups. There is a statistically significant trend towards the lower ranked groups, i.e. firmer stools (*P* = 0.039).

**Table 3:** Bristol Stool Form Scale in the present and a previous study

Bristol Stool Form Scale	Pre-operative *N* = 26	Post-operative *N* = 26	*P* value	Heaton et al [12] *N* = 1897
Not recorded			*P* = 0.028^b^	
*n* (%)	1 (3.8 %)^a^	3 (11.5 %)^a^		-
1. Separate hard lumps, like nuts				
*n* (%)	0	3 (11.5 %)		95 (8.1 %)
2. Sausage-shaped but lumpy				
*n* (%)	2 (7.7 %)	4 (15.4 %)		153 (13.1 %)
3. Like a sausage or snake but with cracks on its surface				
*n* (%)	1 (3.8 %)	3 (11.5 %)		214 (18.4 %)
4. Like a sausage or snake, smooth and soft				
*n* (%)	12 (46.2 %)	8 (30.8 %)		518 (44.5 %)
5. Soft blobs with clear-cut edges				
*n* (%)	8 (30.8 %)	3 (11.5 %)		62 (5.4 %)
6. Fluffy pieces with ragged edges, a mushy stool				
*n* (%)	2 (7.7 %)	1 (3.8 %)		53 (4.5 %)
7. Watery, no solid pieces				
*n* (%)	0.0	1 (3.8 %)		Not assessed

^a^Three patients did not record any bowel motions post-operatively during the 7-day diary, one of whom returned a similar nil record pre-operatively. Discussion with the participants confirmed that these were accurate records in each case (i.e. no bowel motions during the 7 days). As such, this data is included and analysed as zero rather than being excluded from analysis

^b^ Wilcoxon signed rank test

**Fig. 3:**
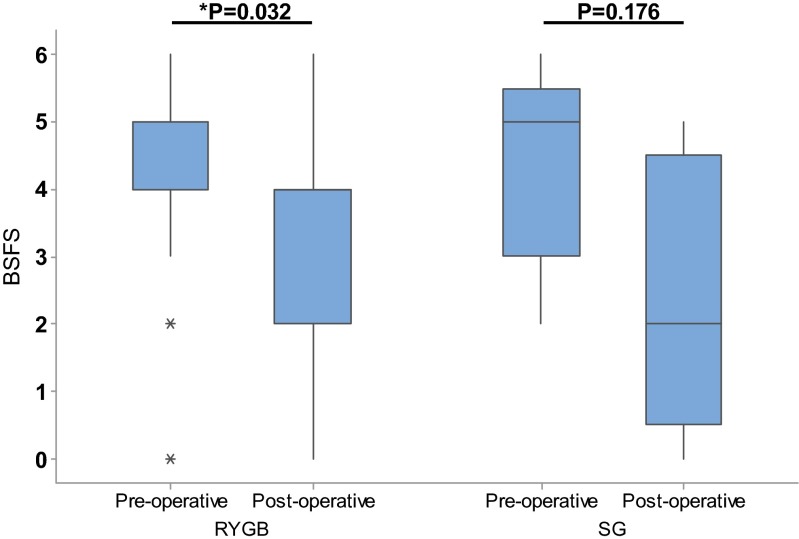
Usual Bristol Stool Form Scale before and after the different bariatric procedures. IGB patients not shown (*n* = 2). *Central line* represents median (BSFS score 4 for RYGB both pre- and post-operatively). *Top* and *bottom lines* of *box* represent 75th and 25th centiles, respectively. *Whiskers* represent range (minimum to maximum). *Represents outliers, defined as at least 1.5 times IQR away from 25th or 75th percentile. The *P* value for RYGB remains significant after exclusions of outliers (*P* = 0.032)

Overall, there was a statistically significant reduction in the mean usual BSFS score (towards firmer stool) from 4.1 (±1.3) pre- to 3.1 (±1.9) post-operatively (*P* = 0.016). Figure 3 shows a statistically significant decrease in usual BSFS score in patients undergoing RYGB (*P* = 0.032), but not for SG (*P* = 0.176) or IGB (*P* = 0.655). There were no significant correlations between the change in BSFS and alimentary limb length (RYGB only), or any of the reported dietary intake measures.

### Constipation, Diarrhoea and Use of Medication

At 6 months post-operatively, the proportion of patients suffering from constipation increased from 8 to 27 %, but this difference was not statistically significant (*P* = 0.125). The use of opiate analgesia did not significantly change pre- to post-operatively, 12 and 8 %, respectively (*P* = 0.317). The use of laxatives increased from 12 % pre- to 19 % post-operatively, but this difference was not statistically significant (*P* = 0.688). None of the patients suffered from diarrhoea pre-operatively, and only one patient reported significant diarrhoea at follow-up. This patient was treated by IGB and concurrently commenced on Orlistat.

### Faecal Incontinence

None of the participants reported faecal incontinence pre- or post-operatively. One participant reported frequent stool seepage/staining pre-operatively (five episodes after eight recorded bowel motions); however, this resolved post-operatively. No other participants reported stool seepage/staining. None of the participants reported use of pads pre-operatively; however, one participant did report using pads post-operatively. Faecal urgency, defined as the inability to postpone bowel motions by at least 15 min, did not change significantly (*P* = 0.796).

## Discussion

This study shows that constipation is a common problem after bariatric surgery with over a quarter of patients affected at 6 months follow-up. There was a significant 33 % decrease in frequency of bowel motions and a change towards firmer stools, suggesting a slowdown in gut transit post-operatively. This effect was observed despite a small non-significant increase in the use of laxatives, which is likely to be ameliorating the problem in those patients. Since dietary fibre intake has major roles in determining stool volume and gut transit times [16, 17], these changes may be explained, at least in part, by the 28 % decrease in intake of dietary fibre we have observed after bariatric procedures. Only 15 % of participants met the recent SACN recommendations of dietary fibre intake for adults post-operatively [15]. This could be due to difficulties in obtaining adequate fibre from the smaller food portions after bariatric procedures.

### Findings in the Context of Other Studies

As highlighted by a systematic review, there is much heterogeneity in the published literature on the effects of bariatric surgery on bowel function [3]. Our finding of decreased stool frequency is in keeping with observations by Foster et al. [9].

In contrast to our findings, Potoczna et al. [7] described a decrease in constipation from 29.4 % pre- to 7.1 % pre-operatively (*P* < 0.001). They also found an increase in the frequency of loose stools (6.3 to 40.5 %) and diarrhoea (1.6 to 5.6 %). In their study, subjects had a 250-cm alimentary limb compared with a mean of 124 cm in the present study. This difference in surgical technique is likely to be a major factor in explaining the difference in findings. Longer alimentary limbs have been associated with an increase in malabsorptive complications, including an increase in diarrhoea symptoms [18]. The limb lengths used in our RYGB technique are unlikely to cause malabsorption [19]. This is supported by the low incidence of post-operative diarrhoea in this cohort.

Furthermore, the timing of assessment of bowel habit after bariatric surgery may be an important factor. Potoczna et al. assessed RYGB patients at a median of 2.1 years (range 4 months to 5.4 years) post-operatively compared to 6.4 months in the present study. However, given the wide range of follow-up periods in the study by Potoczna et al., the authors analysed the impact of follow-up time on bowel habit and concluded that no significant adaptation was occurring over time.

Our finding of lower BSFS post-operatively suggests an increase in WGTT [11]. This effect may be in part due to the increased post-prandial levels of glucagon-like peptide-1 (GLP-1) and peptide-YY (PYY) after bariatric surgery which delays intestinal transit [20–22]. However, an increase in gastric emptying as well as a faster oro-caecal transit time after RYGB have been reported by some [23, 24], whereas others have not found any increase in either gastric emptying [25, 26] or gut transit time [24]. Fibre has an acceleratory effect on transit time [17], and our findings suggestive of an increase in WGTT post-operatively are consistent with the observed fall in fibre intake.

Bariatric procedures can result in vagal nerve injury [27, 28]. The now seldom used total and selective vagotomy caused diarrhoea in a subset of patients [29]. Our RYGB technique involved mobilising the stomach by dissection of the gastrohepatic ligament close to the gastric wall. This preserves the main vagal trunks in a fashion similar to a highly selective vagotomy and makes extensive vagal nerve injury unlikely. This is consistent with the very low incidence of post-operative diarrhoea in this cohort.

### Strengths and Limitations of Study

The small sample size and the use of self-reported data are the limitations of this study. The reliability of self-reported data is a recognised problem in this patient population [30, 31]. In addition, we collected follow-up data at 6 months post-operatively, and dietary adaptation following RYGB is thought to be occurring from 3 months to at least 2.5 years post-surgery [32]. So, the changes which we observed may not represent a new steady state for these patients. Repeated assessments over longer-term follow-up periods would be useful.

The use of a prospective study design with paired observations using validated assessment tools is the major strength of this study. In particular, the availability of data on individual dietary intakes obtained using a validated FFQ is valuable in developing potential explanations for the observed effects on bowel habit. The observed decrease in dietary fibre intake offers a very practical target for interventions to address the observed adverse effects on bowel habit.

## Conclusions

In summary, in obese patients following bariatric surgery, we found decreased bowel motion frequency and reduced BSFS, which are indicative of prolonged intestinal transit time. These observations were associated with reduced dietary fibre intake which is a likely causal relationship. These findings should alert healthcare professionals involved in caring for obese patients undergoing bariatric procedures to this potentially treatable post-operative problem. Future studies into the optimal dietary advice, treatment and possible fibre supplementation in this group are needed.
